# Oxidative Stress Induces Persistent Telomeric DNA Damage Responsible for Nuclear Morphology Change in Mammalian Cells

**DOI:** 10.1371/journal.pone.0110963

**Published:** 2014-10-29

**Authors:** Elisa Coluzzi, Monica Colamartino, Renata Cozzi, Stefano Leone, Carlo Meneghini, Nathan O’Callaghan, Antonella Sgura

**Affiliations:** 1 Department of Science, University of “Roma Tre”, Rome, Italy; 2 CSIRO Food and nutritional Sciences, Nutritional Genomics Laboratory, Adelaide, Australia; Tulane University Health Sciences Center, United States of America

## Abstract

One main function of telomeres is to maintain chromosome and genome stability. The rate of telomere shortening can be accelerated significantly by chemical and physical environmental agents. Reactive oxygen species are a source of oxidative stress and can produce modified bases (mainly 8-oxoG) and single strand breaks anywhere in the genome. The high incidence of guanine residues in telomeric DNA sequences makes the telomere a preferred target for oxidative damage. Our aim in this work is to evaluate whether chromosome instability induced by oxidative stress is related specifically to telomeric damage. We treated human primary fibroblasts (MRC-5) *in* *vitro* with hydrogen peroxide (100 and 200 µM) for 1 hr and collected data at several time points. To evaluate the persistence of oxidative stress-induced DNA damage up to 24 hrs after treatment, we analysed telomeric and genomic oxidative damage by qPCR and a modified comet assay, respectively. The results demonstrate that the genomic damage is completely repaired, while the telomeric oxidative damage persists. The analysis of telomere length reveals a significant telomere shortening 48 hrs after treatment, leading us to hypothesise that residual telomere damage could be responsible for the telomere shortening observed. Considering the influence of telomere length modulation on genomic stability, we quantified abnormal nuclear morphologies (Nucleoplasmic Bridges, Nuclear Buds and Micronuclei) and observed an increase of chromosome instability in the same time frame as telomere shortening. At subsequent times (72 and 96 hrs), we observed a restoration of telomere length and a reduction of chromosome instability, leaving us to conjecture a correlation between telomere shortening/dysfunction and chromosome instability. We can conclude that oxidative base damage leads to abnormal nuclear morphologies and that telomere dysfunction is an important contributor to this effect.

## Introduction

Telomeres are nucleoprotein complexes that protect the ends of linear chromosomes. Their primary role is to maintain chromosome and genome stability by preventing chromosomal ends from being recognised as double-strand breaks and by protecting them from end-to-end fusion and degradation [1].

In humans, telomeric DNA is typically 10–15 Kb and is composed of tandem (TTAGGG)_n_ hexanucleotide repeats [1].

In addition, telomeres interact with a number of proteins that can influence chromosome-end integrity and dynamics [2]. Among them is telomerase, which is a ribonucleoprotein complex that regulates telomere-length maintenance by adding telomeric repeats to the chromosome 3′-end using an RNA template [3]. Telomerase is inactive in somatic cells; however, it is active in 85% of cancer cells. Alternative Lengthening of Telomeres (ALT) is a separate mechanism of telomere length maintenance based on recombination that is active in the remaining 15% of cases [4], [5].

Dysfunctional telomeres are caused by the loss of telomeric repeats or the loss of protection by telomere-associated proteins and are recognised by many DNA damage response proteins. The recognition of dysfunctional telomeres induces a series of events that compromise the proliferative capacity or viability of the cell, including increased levels of recombination at the chromosome ends, altered gene-expression patterns, chromosome fusions, genome instability, growth arrest and cell death [2], [6]–[9].

In somatic cells, after many rounds of cells division, telomeres become critically short due to the end replication problem and are recognised as DNA damage [10]. The rate of telomere shortening can be accelerated significantly by environmental agents (including radiation and chemical agents) that promote the gradual or sudden loss of sufficient repeated sequences necessary to maintain proper telomere structure [11], [12].

Oxidative DNA damage constitutes the majority of DNA damage in human cells [13]. Reactive oxygen species (ROS) are a source of oxidative stress due to the production of single strand breaks [14] either directly or as an intermediate step in the repair of oxidative base modifications [15] anywhere in the genome.

One form of DNA damage induced by oxidative stress is the alteration of DNA bases to species such as 8-oxoguanine (8-oxoG), thymine glycol, and 5-hydroxy-methyluracil. When the 8-oxoG lesion is not repaired correctly, it induces single or double strand breaks (SSBs or DSBs) and GC-TA mutation, which may lead to genomic instability [16].

During oxidative stress, the possibility of 8-oxoG accumulation within telomeres is enhanced by the high incidence of guanine residues in telomeric DNA sequences [17]. Moreover, telomeres are repaired less efficiently than the rest of the genome [18], [19]. The presence of 8-oxoG inhibits telomerase activity and decreases the binding of telomeric proteins to the telomere sequence, leading to the disruption of telomere length, maintenance and function [19]. It is well known that oxidative stress induces single strand breaks in telomeric DNA [17]. Furthermore, the loss of chromosomal end capping has consequences for a wide range of cellular processes including apoptosis, ageing, carcinogenesis [20], [21] and chromosome instability [22], [23].

Chromosome instability related to telomere dysfunction is mediated mainly by the formation of nuclear anomalies such as micronuclei (MN), nucleoplasmic bridges (NPBs) and nuclear buds (NBUDs), which are biomarkers of genotoxic events and chromosomal instability [24], [25].

MN derive from whole chromosomes and chromosome fragments that hang back during anaphase and fail to be included in the daughter nuclei during telophase, while NPBs are nucleoplasmic bridges that originate during anaphase when dicentric chromosomes are pulled to opposite poles of dividing cells [26]. NBUDs have the same morphology as MN, although they are connected to the nucleus by a slight stem of nucleoplasmic material [27]. Recently, Pampalona et al. [25] proposed that most abnormal nuclear morphologies, in particular NPBs, derive from end-to-end fusions affecting chromosomes with critically short telomeres.

Therefore, telomere integrity appears to be a critical element in chromosome stability and telomere shortening, which is induced by an increase in oxidative stress leading to genomic instability and mal-segregation, which both occur very frequently in tumour cells [28].

Considering that acute oxidative stress can occur in several states, such as physical activity, inflammation, the tumour microenvironment and cells exposed to radiotherapy, in this work, we aimed to study acute oxidative stress-induced damage specifically in the telomeric region through the analysis of telomeric and genomic base (8-oxoG) modification. Moreover, we sought to understand the role of telomeres in the genomic instability observed after treatment with acute oxidative stress. Furthermore, we studied the relationship between telomere length and genomic instability by analysing telomere length and abnormal nuclear morphologies (NPBs, NBUDs and MN).

## Materials and Methods

### Cell and culture conditions

Human primary fibroblasts MRC-5, derived from embryonic human lung primary culture (ECACC, UK), were grown in modified Eagle’s medium (MEM) (Euroclone, Italy) supplemented with 10% foetal bovine serum (Euroclone, Italy), 10,000 units/ml penicillin and streptomycin 10 mg/ml (Biological Industries, Israel), 1% L-Glutamine and 1% non-essential amminoacids (Euroclone, Italy), at 37°C in 95% air and 5% CO_2_ incubator.

Cells were sown on plates 48 hrs before treatment at the density of 3×10^5^ cells per culture flask. Subconfluent cells were treated with H_2_O_2_ (10vol-3%), in a complete medium, at the final concentration of 100–200 µM for 1 hr at 37°C in 95% air and 5% CO_2_ incubator. Cells examined after treatment with H_2_O_2_ were compared to parallel cultured control cells grown in the medium without H_2_O_2_.

### Standard and FPG-modified alkaline comet assay

MRC-5 cells were analysed by alkaline comet asssay as described by Festa et al. [29] to verify the entity of DNA damage induced by H_2_O_2_ treatment. After 1 hr treatment, cells were immediately subjected to the assay (time 0) or grown in fresh medium for different recovery times 1, 4, 15 and 24 hrs to evaluate the activation of repair pathways over time and then subjected to the alkaline comet assay. At the end of treatment, before performing comet assay, an aliquot of cells from each sample was dyed with trypan blue in order to evaluate the percentage of cell death (blue stained cells). This percentage never exceeded 20% in all experimental conditions.

In addition we used formamidopyrimidine-DNA-glycosylase (FPG)-modified comet assay to evaluate oxidative DNA damage. FPG is a glycolase that recognizes and specifically cuts the oxidised bases principally 8-oxoG from DNA, producing apurinic sites converted in breaks by the associated AP-endonuclease activity. Therefore, these breaks can be detected by comet assay and give a measure of oxidative DNA damage, enabling us to detect moderate, but still appreciable damage. We followed the procedure described by Collins et al. [30], with minor modifications. The analysis was performed using a FPG FLARE module (Trevigen, Gaithersburg, MD). Within the module, the manufacturer provided all the reagents used.

Slides were analysed using a fluorescence microscope (Leica) equipped with a camera. Seventy comets on each slide, and two slides for each experimental point, coded and blindly scored, were acquired using “I.A.S.” software automatic image analysis system purchased from Delta Sistemi (Rome-Italy). To quantify the induced DNA damage we used the Tail DNA (TD%), which is a measure of the percentage of migrated DNA in the tail [31]. Experiments were repeated at least three times.

### FPG-sensitive sites within telomeric DNA

400 ng of gDNA was incubated with 12 units of FPG in 1x NEB Buffer #1. A control incubated with 1X NEB Buffer #1 but without FPG (replaced with H_2_O) was also prepared. All samples were set up on ice then incubated at 37°C overnight to allow exhaustive digestion to occur. Five 84-mer oligomers of TTAGGG repeats were designed containing either zero, one, two, four, or eight 8-oxoG bases incorporated into the telomeric tandem repeat sequence. A reverse oligomer was used (an 84-base sequence of 14 CCCTAA repeats) to construct duplex substrates. All oligomers were sourced from GeneWorks (Adelaide, Australia).

The quantitative Real-Time amplification of the oligomers was performed as described by O’Callaghan et al. [32], [33]. Breifly, each 20 µL qPCR reaction was composed as follows: 40 pg of digested or undigested oligomer DNA or 40 ng of digested or undigested genomic DNA, 1xSYBR Green master mix (Applied Biosystems [AB] Foster City, CA, USA), 100 nM telo1 forward, 100 nM telo2 reverse primers [32]. All samples were run on an ABI 7300 Sequence Detection System with the SDS Ver. 1.9 software (Applied Biosystems [AB] Foster City, CA, USA). Each sample was analysed in triplicate. Cycling conditions were: 10 minutes at 95°C, followed by 40 cycles of 95°C for 15 seconds and 60°C for one minute. For the 36B4 amplicon we also used 4 ng of digested or undigested genomic DNA, 1xSYBR Green master mix, 100 nM 36B4 forward, 100 nM 36B4 reverse primers as described in O’Callaghan et al. [32]. All samples were run on an ABI 7300 Sequence Detection System with the SDS Ver. 1.9 software (Applied Biosystems [AB] Foster City, CA, USA). Each sample was analysed in triplicate. Cycling conditions were: 10 minutes at 95°C, followed by 40 cycles of 95°C for 15 seconds and 60°C for one minute.

Following qPCR amplification profiles of FPG treated and untreated samples were compared. A ΔCT (CT treated - CT untreated) was then calculated. The number of 8-oxoG in each oligomer was then translated into the number of 8-oxoG bases per kb telomeric DNA through a standard curve established as outlined in O’Callaghan et al. [33].

### Collection of chromosome spreads and Quantitative-Fluorescence “in situ” Hybridization Analysis (Q-FISH)

After treatment, PBS washed out media and cells were trypsinized and sown at the density of 3×10^5^ cells per culture flask and left to grow in complete medium for 24, 48, 72 and 96 hrs and collected.

Chromosome spreads were obtained following 30 min incubation in 30 µM calyculin-A (Wako, Germany), [34]. Spreads of these prematurely condensed chromosomes (PCC) were prepared by a standard procedure consisting of treatment with a hypotonic solution (75 mM KCl) for 28 min at 37°C, followed by fixation in freshly prepared Carnoy solution (3∶1 v/v methanol/acetic acid). Cells were then dropped onto slides, air dried, and utilized for cytogenetic analysis.

The Q-FISH tecnique was based on the use of peptide nucleic acid (PNA) telomere oligonucleotides, that generate stronger and more specific hybridization signals than the same DNA oligonucleotides. The resolution of Q-FISH was in the region of about 200 bp [35]. The Q-FISH allowed: (i) precise measurement of individual telomeres at every single chromosome arm (ii) to detect even small differences in telomere length [36]. Q-FISH staining was performed as previously described by Berardinelli et al. [37] with minor modifications. Briefly slides and probes (Cy3 linked telomeric, PANAGENE, Korea and chromosome 2 centromeric Peptide Nucleic Acid PNA probes DAKO Cytomatation, Denmark) were co-denatured at 80°C for 3 min and hybridized for 2 hrs at room temperature in a humidified chamber. Slides were counterstained with 4,6-diamidino-2 phenylindole (DAPI, Sigma Aldrich, St. Louis, USA) in Vectashield (Vector Laboratories, Burlingame, CA). Q-FISH technique required appropiate digital image analysis system designed to control acquisition of digital images, as well as to perform fluorescence intensity measurement [35]. However, Q-FISH is a complex technique and it requires proper calibration protocols to eliminate inherent variations associated with fluorescence microscopy [38]. In our laboratory we use centromere of chromosomes 2 as the internal reference in each metaphase analysed. Images were captured at a 63X magnification with an Axio Imager M1 (Carl Zeiss, Germany) equipped with a CCD camera. The telomere size was analysed with ISIS software (MetaSystems, Germany). In particular the software calculates telomere lengths as the ratio between the total telomeres fluorescence (T) and the fluorescence of the centromere of the two chromosomes 2 (C). Data were expressed as a percentage (T/C%) [36]. Experiments were repeated at least three times, and at least 50 metaphases were scored for each experiment.

### RTQ-PCR TRAP Assay

The SYBR green RTQ-TRAP assay was conducted as described elsewhere [39] with minor modifications. The reaction was performed with protein extracts, telomerase and anchored return primer mixed with SYBR Green PCR Master Mix (Biotools, Spain). The reaction was performed by the RotorGene 6000 Thermal cycler (Corbett, Australia). The threshold cycle values (C_t_) were determined from semi-log amplification plots (log increase in fluorescence as a function of cycle number) and compared with standard curves generated from serial dilutions of telomerase-positive (tel^+^) cell extracts (human large cell lung cancer H460). Each sample was analysed in duplicate, and the experiment was representative. Telomerase activity was expressed relative to the telomerase-positive (tel^+^) sample.

### Co-FISH (Chromosome Orientation-FISH) analysis

After 1 hr treatment, cells were trypsinized and sown at the density of 3×10^5^ cells per cultured flask. Cells were incubated at 37°C in the presence of 5′-bromo-2′-deoxyuridine (BrdU; Sigma Aldrich, St. Louis, USA) at a final concentration of 2.5×10^−5^ M 24 hrs before fixation. 5×10^−6^ µM colchicine was added during the last 5 hrs of incubation. Cells were then collected and metaphase spreads prepared as yet described. CO-FISH, a recombination-based mechanism that analysed the telomere recombination events between sister chromatids, was performed as previously described by Berardinelli et al. [39]. We used first a (TTAGGG)_3_ probe labelled with Cy3 and then a (CCCTAA)_3_ probe labelled with FITC (Panagene, Korea). Images were captured with an Axio Imager M1 (Carl Zeiss, Germany) equipped with a CCD camera (MetaSystems, Germany). Only T-SCE (Telomere Sister Chromatid Exchanges) events observed with both leading (Cy3) and lagging (FITC) strand probes simultaneously were considered positive. We calculated the T-SCE ratio as the ratio of the frequency of treated samples relative to the frequency in control samples. A total of 1500 chromosomes were scored.

### Cell growth and viability assay

Cells were harvested by trypsinization and resuspended in 5 ml of complete medium. Total cells were diluted (1∶20) in 0.9% NaCl and enumerated using a haemocytometer (Coulter Counter). Percentage of dead cells was determined by flow cytometer (Galaxy, DAKO Cytomatation, Denmark) with propidium iodide permeability exclusion test. Briefly, cells suspension were incubated 5 minutes with 15 µg/ml of propidium iodide and then analyzed on FL3 parameter in log scale. The percentage of propidium-positive cells were determined by an opportune electronic marker. Every data point was assessed in triplicate.

### Cytokinesis-block micronucleus assay

After 1 hr treatment cells were plated on petri dish at different density and Bi-nucleated (BN) MCR-5 were obtained adding cytocalasin B (1 mg/ml stock solution in dimethyl sulphoxide; Sigma Aldrich, St. Louis, USA) at a final concentration of 3 µg/ml 24 hrs before fixation [40]. The culture was fixed in freshly prepared Carnoy solution (3∶1 v/v methanol/acetic acid) and afterward dyed with DAPI (Sigma Aldrich, St. Louis, USA) in Vectashield (Vector Laboratories, Burlingame, CA).

The analysis of abnormal nuclear morphologies was performed on 1000 BN cells.

Experiments were repeated at least three times.

### Scoring criteria

A BN cells were scored as previously described by Fenech [41]. Briefly, doughter nuclei must be situated in the same cytoplasm, had approximately the same size, staining pattern and staining intensity. MN was considered as a small nucleus, morphologically identical to the cell nucleus. NBUD was considered a small nucleus but connected to the cell nucleus. NPB was considered to be the DNA connection between the two doughter nuclei [26].

### Data analysis

The statistical tests vary according to the technique used. We performed the Mann-Whitney U test for the statistical analysis of the telomere length (Q-FISH) and the SSBs, measured by the comet assay. We used the binomial probability test for the statistical analysis of the NPBs, MN and NBUDs and CO-FISH analysis. We used the t-test (2 way ANOVA) for the analysis of telomerase activity, for the analysis of telomeric damage and for the comparisons among multiple group in cell growth and viability assay. Significance was accepted for value p<0.05.

## Results

### Oxidative stress induces oxidative base damage that does not persist 24 hrs after treatment

The standard alkaline comet assay is a sensitive and reliable method used to investigate cellular DNA damage, in particular, SSBs and DSBs.

**Figure 1:**
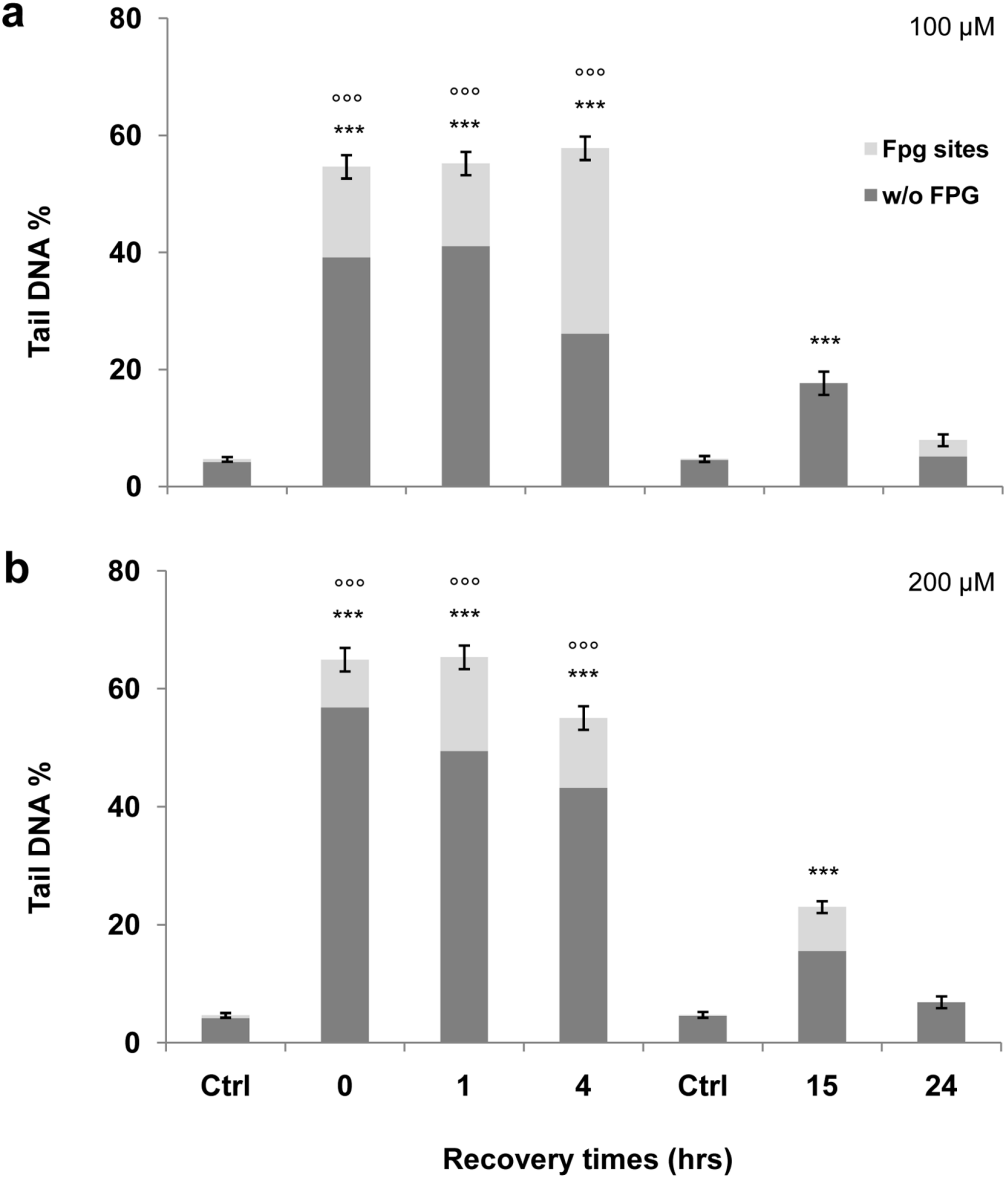
Standard and FPG-modified alkaline comet assay. Standard and FPG-modified versions of the comet assay have been used to evaluate the fold increase in genomic damage induced by hydrogen peroxide with respect to the control value. The net cleavage sites generated by FPG activity were calculated subtracting the value of total DNA damage yielded by the samples not treated with the enzyme from the DNA damage value obtained from samples treated with the enzyme. The value of this subtraction is shown in the graph as a light grey portion labelled ‘‘FPG sites.’’ To assess any changes in Tail DNA values, we analysed control cells immediately (time 0) and after 15 hrs treatment. All values are normalised to the control values. A significant increase (p<0.001) is observed in the percentage of DNA damage immediately after treatment (time 0). DNA damage increases in a dose dependent manner after 1 hr treatment. We observed a time-dependent decrease of genomic damage to the control value 15 and 24 hrs after treatment. Error bars denote the standard error. (a) DNA damage induction by 100 µM H_2_O_2_. (b) DNA damage induction by 200 µM H_2_O_2_. ***p<0.001; **p<0.01; *p<0.05. Treated cells plus FPG versus controls plus FPG by Mann–Whitney U test. °°°p<0.001; °°p<0.01; °p<0.05 Treated cells plus FPG versus cells of the same experimental point without FPG by Mann–Whitney U test.

The most frequent DNA damage induced by oxidative stress is base modification, and the principal product is 8-oxoG [42]. For this reason, we performed the standard comet assay together with its FPG-modified version, which is more sensitive to the presence of oxidised bases. The standard comet assay revealed a statistically significant increase (p<0.001) in DNA damage with 100 µM H_2_O_2_ (Fig. 1a) and with 200 µM H_2_O_2_ (Fig. 1b) compared to the untreated control immediately after treatment (time 0), 1 hr and 4 hrs after treatment. The DNA damage observed in the FPG-modified comet assay (grey portion of the columns) was statistically larger (p<0.001) than that from the standard comet assay immediately (time 0), 1 hr and 4 hrs after treatment with 100 and 200 µM H_2_O_2_. Genomic damage decreased 15 hrs after treatment, although it persisted significantly higher than the control value. At 15 hrs, Tail DNA values obtained from the FPG-modified assay are not statistically different from those produced by the standard version of the comet assay for both doses used. A complete repair of genomic damage was observed 24 hrs after treatment both with the standard and FPG-modified comet assay, as evidenced by the similar values yielded by the treated samples and the controls.

### Oxidative stress induces oxidised telomeric DNA that persists 24 hrs after treatment

The high incidence of guanine residues in telomeric sequences makes these more susceptible to oxidative damage, especially to the accumulation of oxidised bases such as 8-oxoG. A new technique that uses quantitative PCR (qPCR) with telomere-specific primers is an extremely sensitive method to measure the amount of oxidised residues by analysing the abundance of telomeric FPG-sensitive sites within telomeric DNA [33].

**Figure 2:**
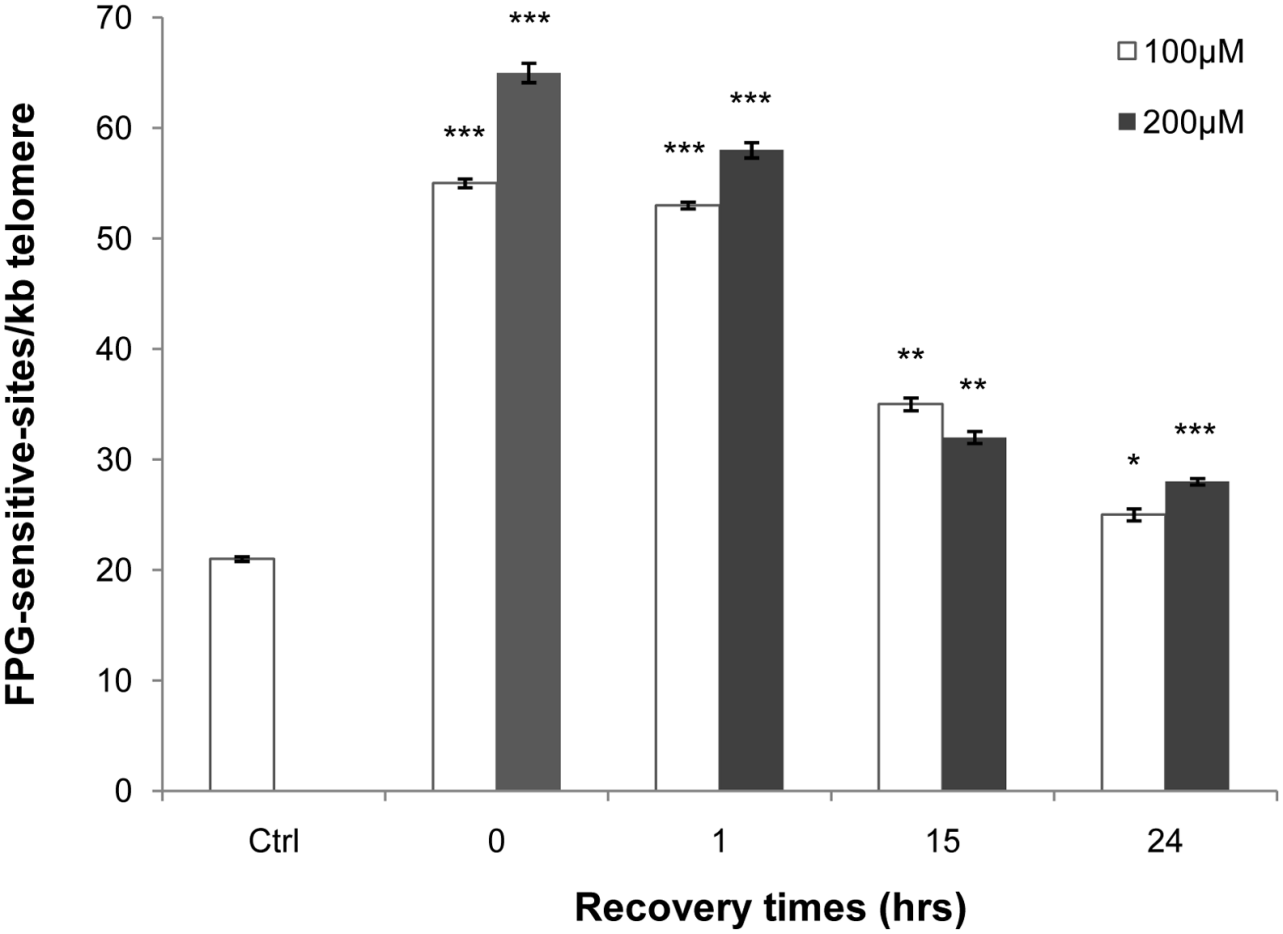
FPG-sensitive sites within telomeric DNA. Quantitative PCR was used to evaluate the amount of FPG-sensitive sites within telomeric sequences after 1 hr treatment with hydrogen peroxide. The columns denote the results obtained from two different doses of H_2_O_2_ (100 and 200 µM). We observed a significant increase (p<0.001) in sensitive sites immediately after treatment (time 0) for both doses. At subsequent recovery times (1, 15 and 24 hrs), we observed a time-dependent decrease of telomeric damage that persists at a value significantly higher than the control value. Specifically, we observed significant persistent telomeric damage after 24 hrs of recovery, especially at 200 µM H_2_O_2_. Error bars denote the standard error. Statistical analysis was performed between treated and control samples. *p<0.05, **p<0.01, ***p<0.001 by Student’s t-test.

We treated cells with either 100 or 200 µM H_2_O_2_ for 1 hr and collected them immediately (time 0), 1, 15 and 24 hrs after treatment. For both 100 and 200 µM H_2_O_2_ treatments, we observed a dose-dependent increase of telomeric damage immediately after treatment (p<0.001) that decreased with time up to 24 hrs of recovery (Fig. 2). However, it is interesting to note that a significant amount of telomeric damage (p<0.001) persists 24 hrs after treatment for both doses.

### Oxidative stress induces temporary telomere shortening

**Figure 3:**
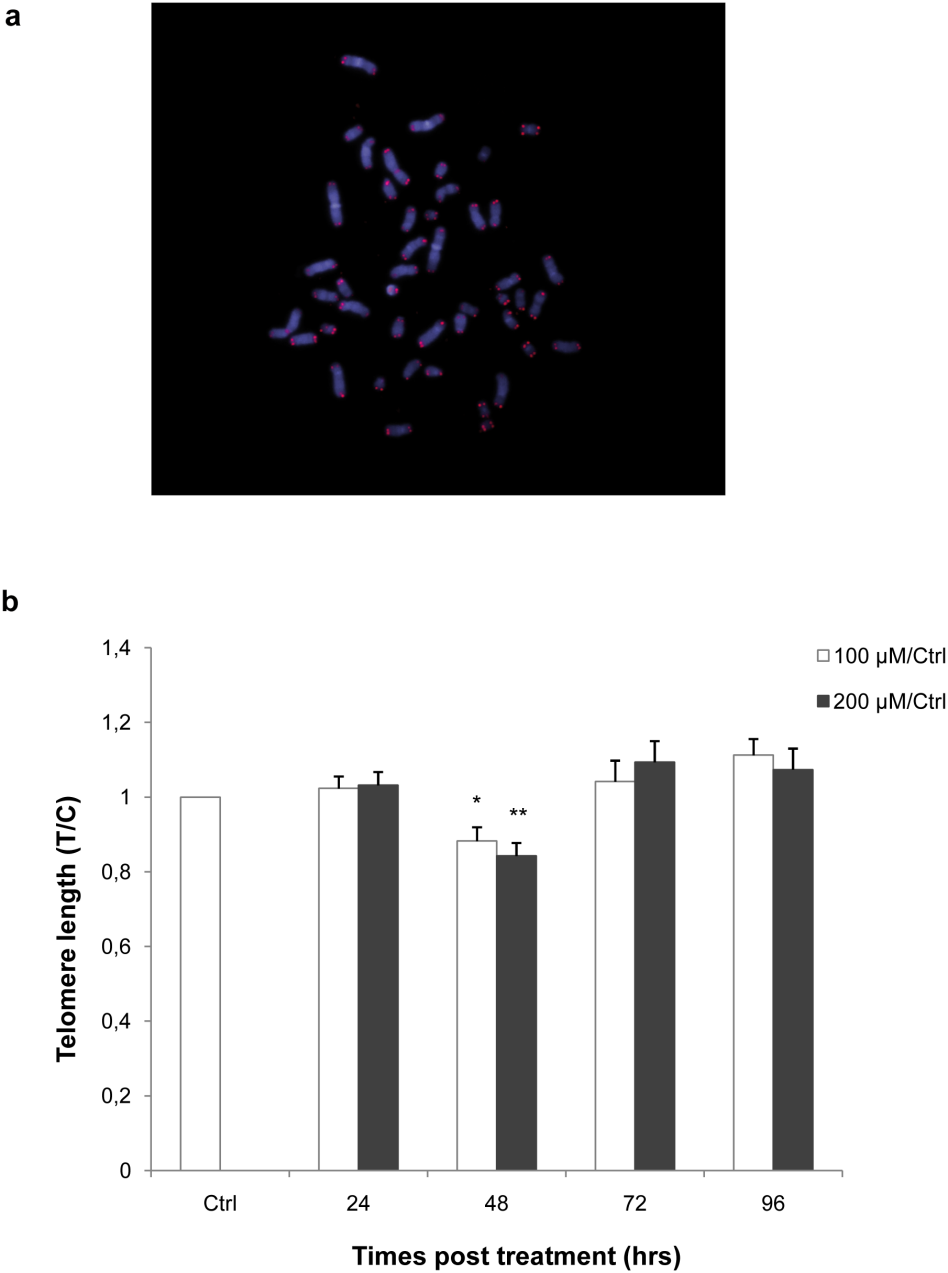
Quantitative-Fluorescence *in* *situ* Hybridization analysis. a) Example of cell stained by Q-FISH at metaphase to measure the telomere length. b) The histogram represents results obtained by the analysis of telomere length after hydrogen peroxide treatment. Telomere lengths (T/C) were calculated as the ratio between the total telomeres fluorescence (T) and the fluorescence of the centromere of the two chromosomes 2 (C), used as the internal reference in each metaphase analysed; data obtained from treated samples were normalised to the average ratios measured on the control sample at the same times post treatment. The values less than 1 (control value) represent a significant (100 µM, p<0.05; 200 µM, p<0.01) reduction of the telomere length 48 hrs after treatment. Conversely, values at other times were no different than the control. Error bars were calculated using standard error propagation rules. Statistical analysis was performed between treated and control samples. *p<0.05; **p<0.01 by Mann-Whitney U test.

We performed telomere length analysis (Fig. 3a) to test whether our treatment induced telomere shortening, as previously demonstrated by von Zglinicki et al. [17]. The kinetics of telomere length are shown in Fig. 3b. We observed no change in telomere length 24 hrs after treatment, while significant telomere shortening was observed 48 hrs after either dose of hydrogen peroxide (100 µM, p<0.05; 200 µM, p<0.01). The telomere length reduction was approximately 12% and 16% for treatments with 100 µM and 200 µM H_2_O_2_, respectively. Normal telomere length was restored 72 hrs after treatment and did not change for up to 96 hrs.

### Hydrogen peroxide does not induce telomerase activity as evaluated by RTQ-PCR TRAP assay

**Figure 4:**
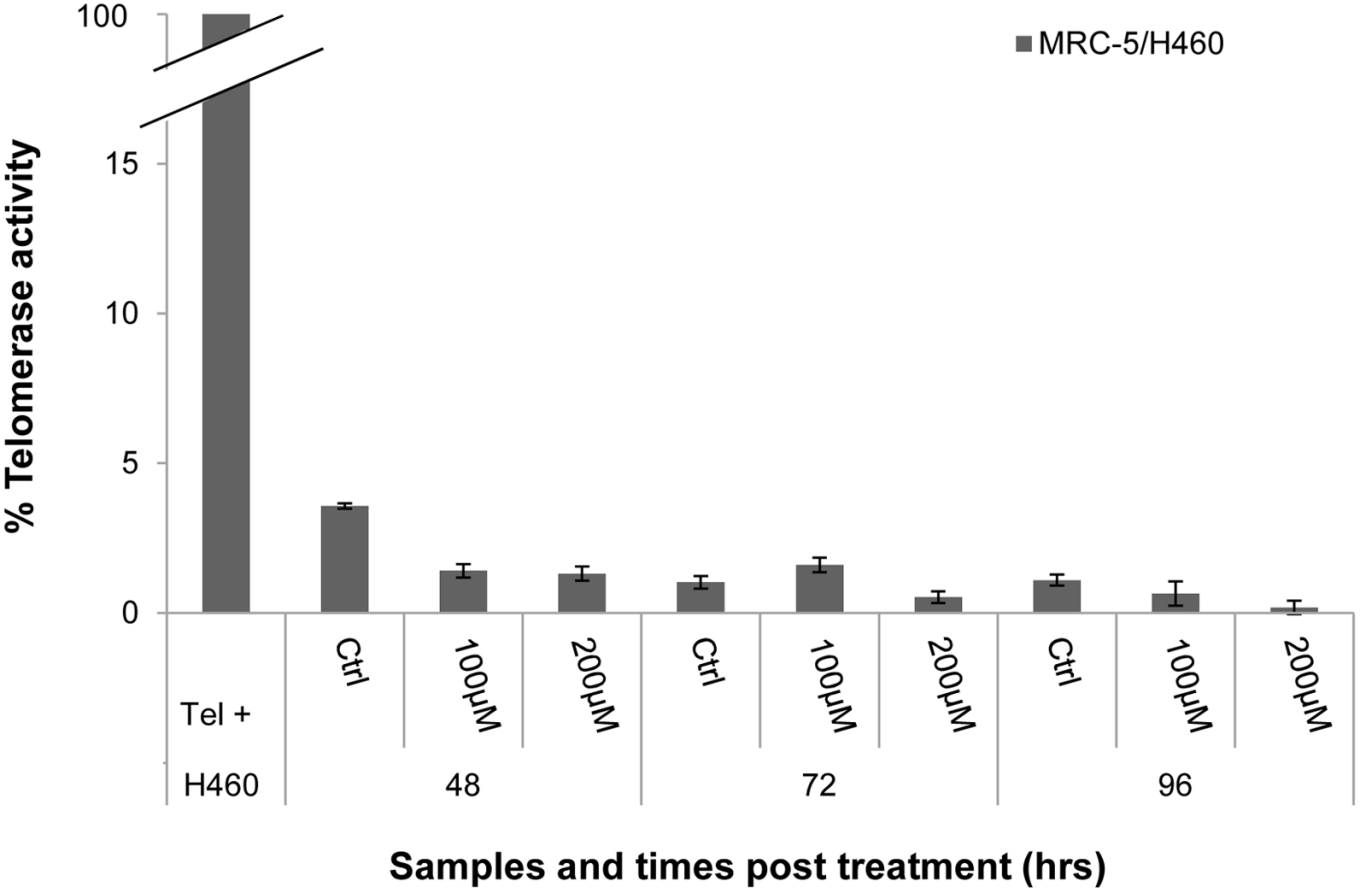
RTQ-PCR TRAP assay. The graph displays data on telomerase activity. Data were normalised to the control value represented by the tumour cell line H460, which is considered to have 100% telomerase activity. Our samples yielded values definitively less than 5%, indicating no telomerase activity in our samples at different doses and times post treatment. The error bars were calculated using standard error propagation rules. p>0.05 by Student’s t-test.

To evaluate if the telomere restoration observed at longer recovery times could be telomerase-dependent, telomerase activity was measured by RTQ-PCR TRAP assay (Fig. 4) [43]. We compared our samples with the strong telomerase-positive tumour cell line H460, which is assigned 100% telomerase activity. The results demonstrated no telomerase activity for either dose and for all times post treatment, confirming that H_2_O_2_ did not induce telomerase activation.

### Hydrogen peroxide does not induce sister chromosome exchanges

**Figure 5:**
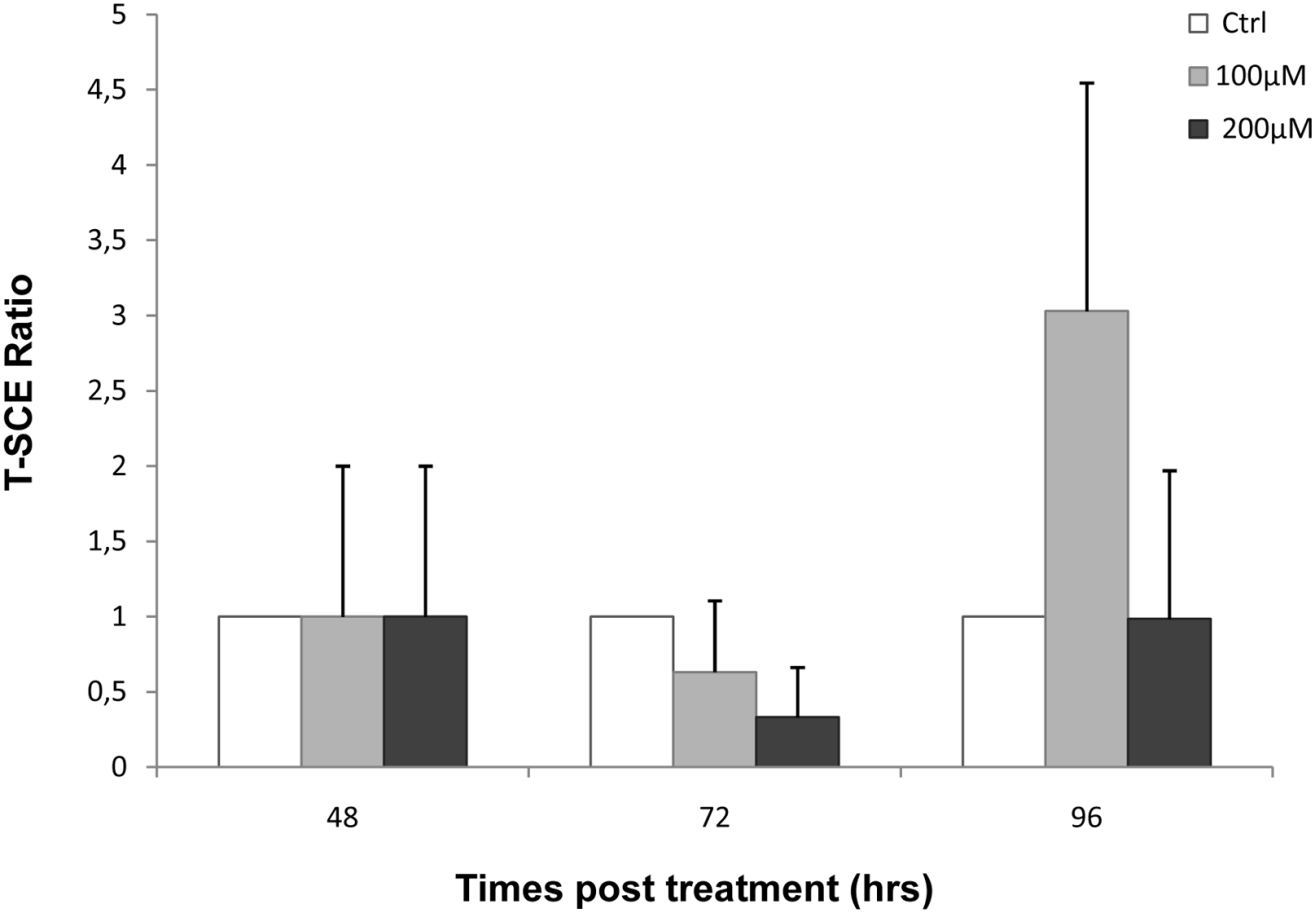
CO-FISH (Chromosome Orientation-FISH) analysis. The histogram represents results obtained by T-SCE (Telomere Sister Chromatid Exchanges) analysis in MRC-5 cells after hydrogen peroxide treatment. The T-SCE ratio was calculated as the ratio of the frequency from treated samples to the frequency from control samples. The results revealed no significant differences between treated and control samples. The error bars denote the standard deviation (± SD). p>0.05 by binomial probability test.

To demonstrate that the telomere restoration observed at 72 and 96 hrs post-treatment in human primary fibroblasts is not due to the activation of the ALT pathway, we performed CO-FISH analysis. The results revealed no statistical differences in T-SCE frequency at these times after oxidative stress (Fig. 5), indicating that the ALT pathway is not induced by H_2_O_2_.

### Oxidative stress induces a decrease in cell growth rate

**Figure 6:**
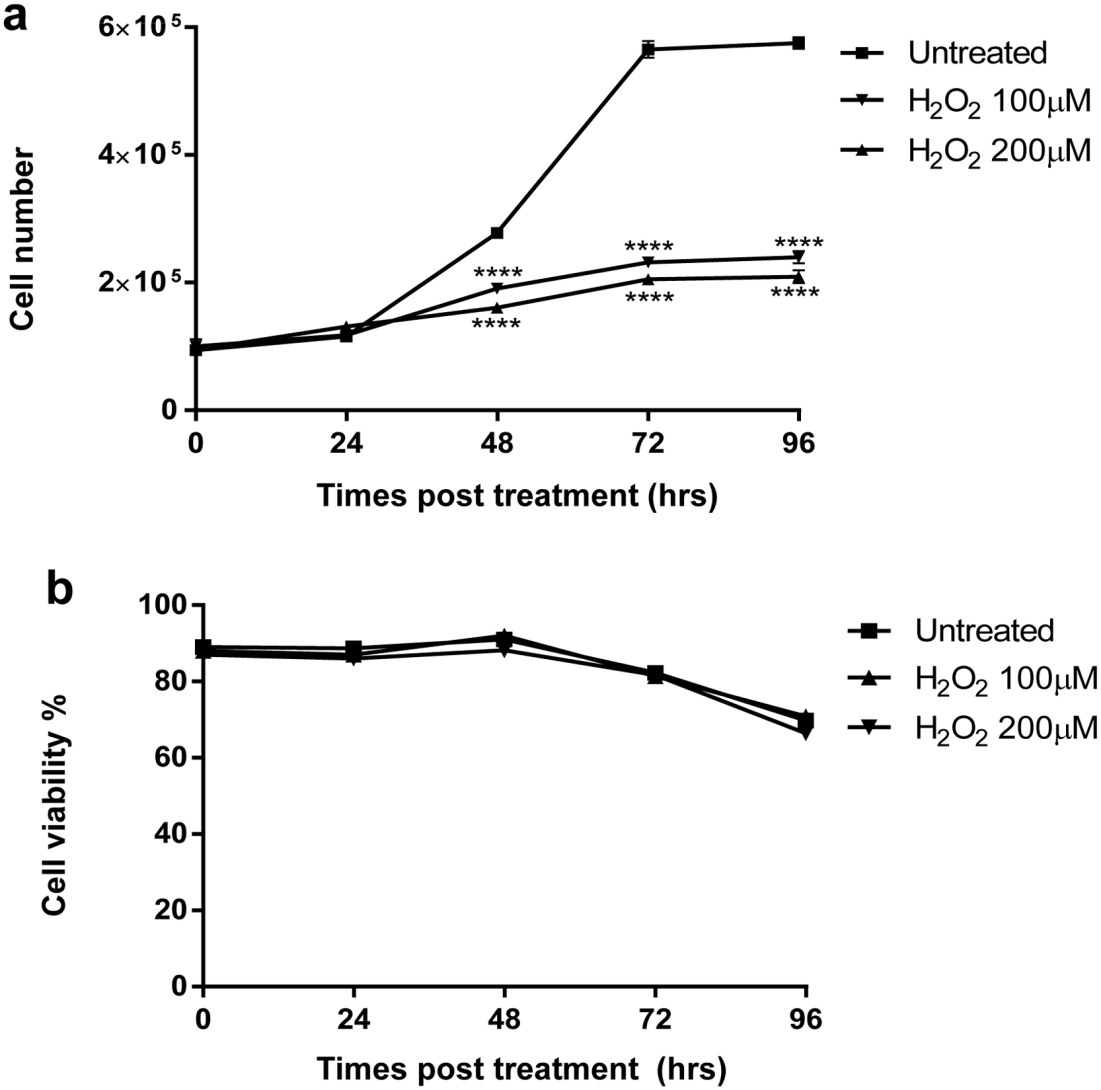
Cell growth and viability assay. MRC-5 cells were treated with H_2_O_2_ at 100 and 200 µM. After treatment, cells were seeded (t_0_) and harvested at different times. Total cells were counted using an electronic haemocytometer (a), and the percentage of viable cells was determined by propidium iodide exclusion by flow cytometry analysis (b). Every data point was assessed in triplicate and data are presented as means ± SD. Comparisons between multiple groups were made by a two-way analysis of variance (ANOVA). ****p<0.0001.

To evaluate if telomere length restoration could be attributed to a cellular selection system that favours cells with longer telomeres, we performed a cell growth curve (Fig. 6a). For both 100 and 200 µM hydrogen peroxide treatments, we observed a significant growth delay starting at 48 hrs (p<0.0001) and persisting up to 96 hrs. The log phase doubling time switches from 21 hrs for untreated cells to 30 and 37 hrs for 100 and 200 µM hydrogen peroxide-treated cells, respectively, revealing a great delay of cell growth. Cell viability was not different between treated and untreated cells (Fig. 6b). As previously demonstrated by Baglole et al. [44], we observed slight propidium iodide cell permeability in both adherent and suspension cells.

### Abnormal nuclear morphology (ANM) induction is a measure of chromosome instability induced by oxidative stress

**Figure 7:**
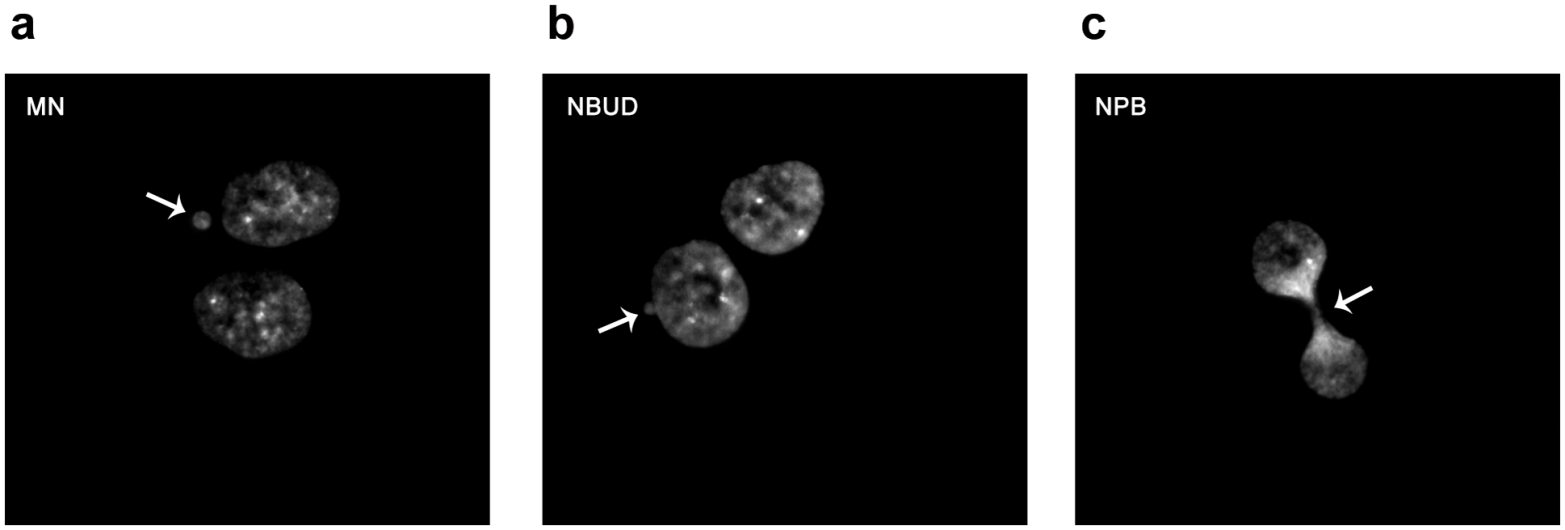
Example of chromosome instability biomarkers. Representative images of Bi-nucleate (BN) cells with different abnormal nuclear morphologies. The arrows denote: (a) Micronuclei (MN), (b) Nuclear Bud (NBUD), (c) Nucleoplasmic Bridge (NPB).

**Figure 8:**
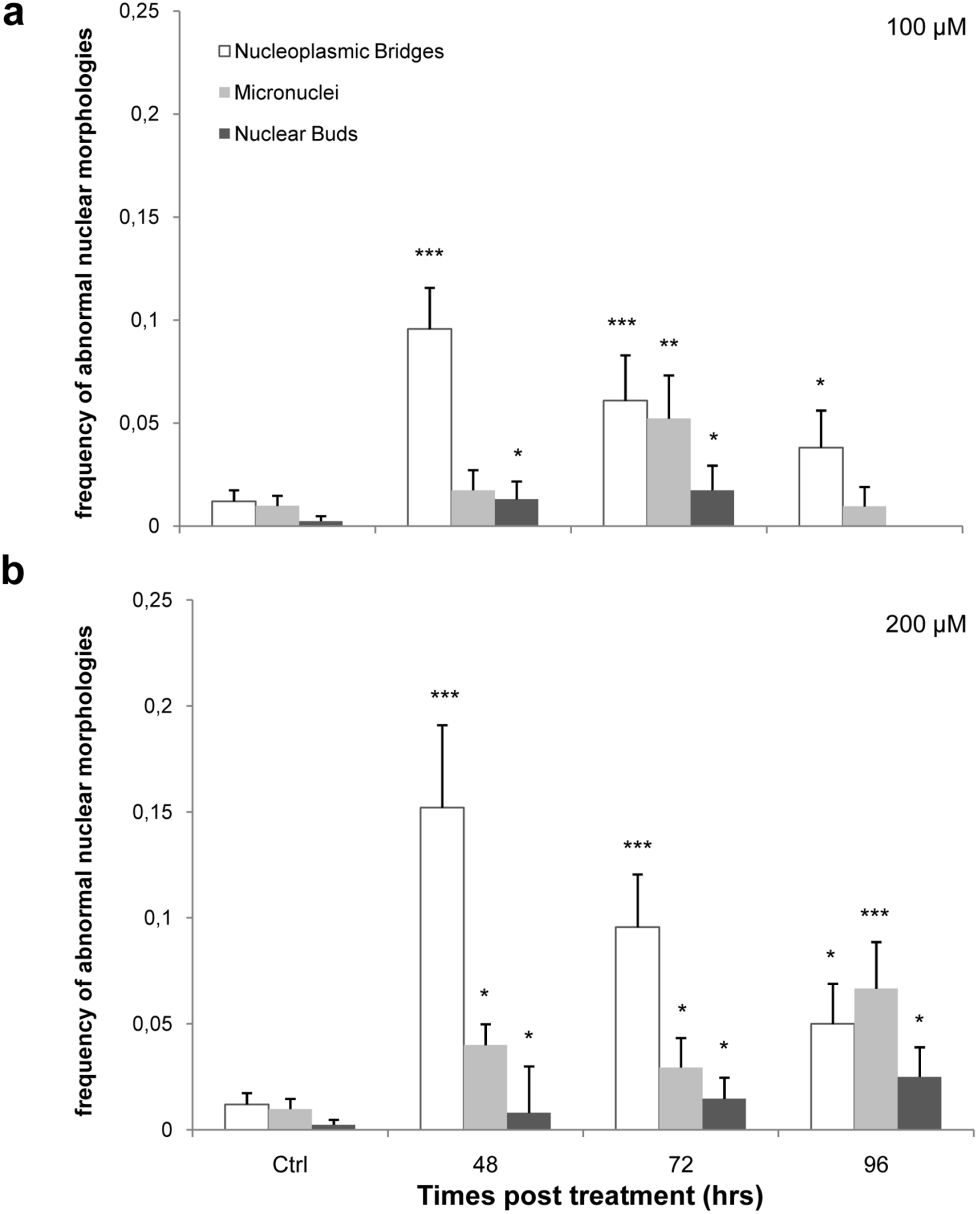
Abnormal Nuclear Morphologies. The different columns denote the abnormal nuclear morphologies analysed (NPBs, MN and NBUDs) at different times, compared to the control data. (a) Data obtained with 100 µM H_2_O_2_. We observed a significant increase of NPBs 48 hrs after treatment and a decrease at subsequent times. For MN and NBUD, we observed a fluctuating trend. (b) Data obtained with 200 µM H_2_O_2_. We again observed a significant increase of NPBs 48 hrs after treatment and a decrease with time until 96 hrs. The frequencies of MN and NBUDs exhibited a time-dependent increase up to 96 hrs. Error bars denote standard error. *p<0.05; **p<0.01; ***p<0.001 by a binomial probability test.

The main goal of this work was to assess the role of telomeres on chromosome instability (CIN). To achieve this aim, we analysed MN, NBUDs and NPBs [26], [24] (Fig. 7a, b, c), biomarkers of chromosome instability that are considered telomere dysfunction-dependent cytogenetic end-points, as a function of H_2_O_2_ doses and times (48, 72 and 96 hrs after treatment) (Fig. 8).

The results revealed a dose-related increase of ANMs 48 hrs after treatment for both 100 (Fig. 8a) and 200 µM (Fig. 8b) doses. In particular, we observed a significant increase in the frequency of NPBs (p<0.001) at 48 hrs and a decrease at subsequent times.

For biomarkers MN and NBUDs, we observed a fluctuating trend for 100 µM hydrogen peroxide (Fig. 8a); conversely, at the higher dose, MN and NBUDs displayed a significant time-dependent increase (Fig. 8b).

### Oxidative stress induces an inverse relationship between telomere length and abnormal nuclear morphologies

NPBs have been shown to correlate with telomere defects [24], [25].

**Figure 9:**
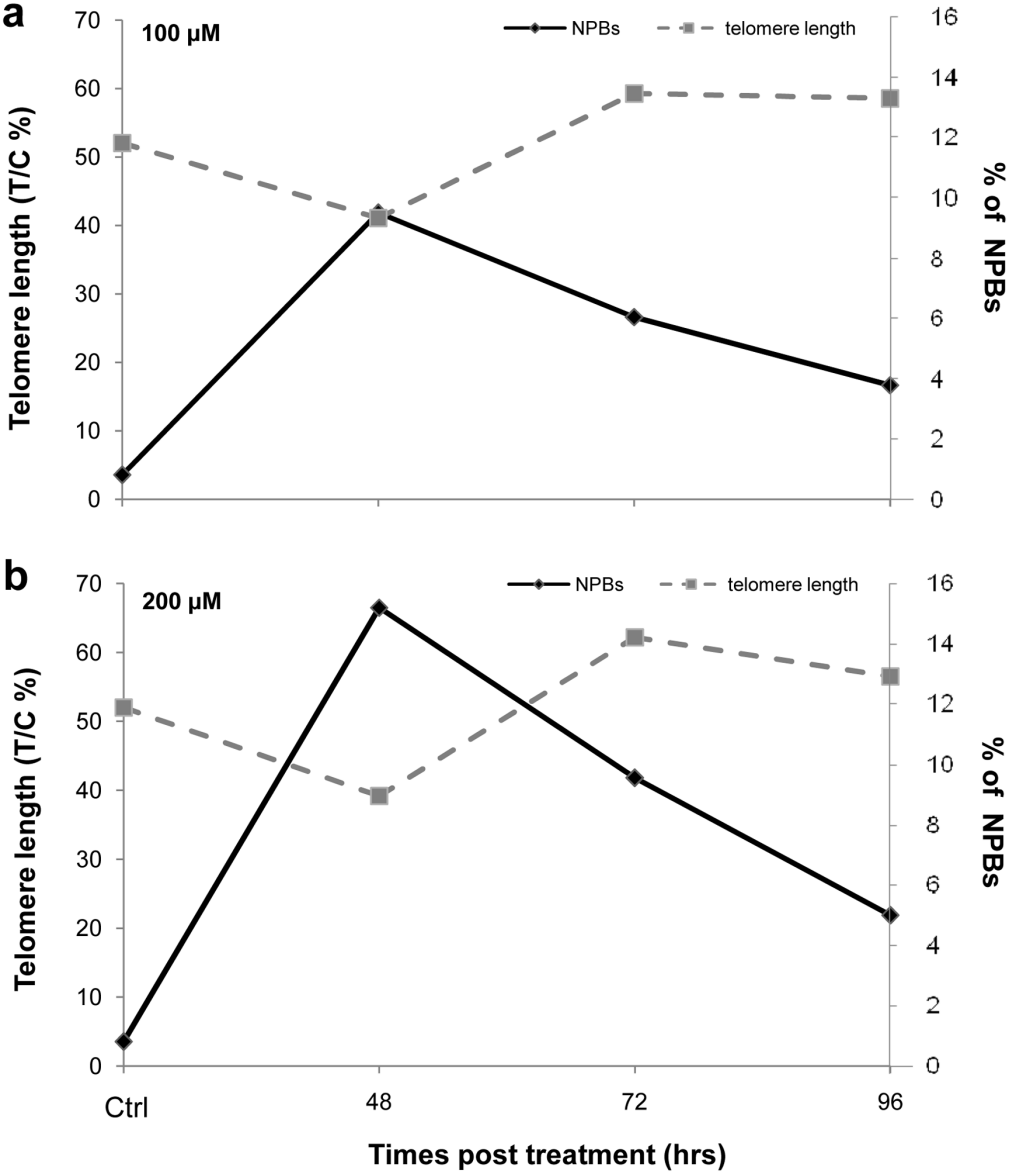
CIN-telomere length. The graphs indicate the relationship between telomere length, expressed as T/C%, and the % of NPBs in 1000 bi-nucleated cells at both doses (a) 100 µM; (b) 200 µM. For both doses, we observed a significant decrease of the T/C% and an increase of the % of NPBs 48 hrs after treatment. The other times revealed a restoration of telomere length to the control value and a decrease in the % of NPBs.

Given that our work has demonstrated that oxidative stress induces telomere shortening and ANM, we decided to study the specific correlation between telomere length and NPBs by comparing results obtained from Q-FISH (Fig. 3b) and NPBs analysis (Fig. 8) for each dose and for all recovery times. The results are shown in Fig. 9. Notably, the lowest telomere length corresponds to the highest level of chromosome instability for both 100 and 200 µM H_2_O_2_ (Fig. 9a and 9b, respectively). At the longer times, we observed the restoration of telomere length and a decrease in the frequency of NPBs.

## Discussion

Telomeres are the nucleoprotein structures that protect the ends of linear chromosomes, preserving genome and chromosome stability [1]. Oxidative stress damages DNA, especially telomere structure [45], [46]. Oxidative stress that produces ROS has been shown to accelerate telomere shortening in replicating fibroblasts in vitro [47]. Other studies have shown accelerated telomere shortening in cells from patients with mutations in mitochondrial DNA characterised by an increased production of reactive oxygen species [48]. The evidence causally linking reactive oxygen species with telomeres is derived from experiments in which oxidative stress induced by arsenic yields telomere attrition, chromosome instability and apoptosis [49]. However, it is unclear how oxidative DNA damage compromises telomere length and integrity. It was hypothesised that telomere sequences, rich in guanine residues, are more susceptible to oxidative stress, mainly by the formation of 8-oxoG [18]. Furthermore, these base modifications could lead to single strand breaks, leading to the loss of the distal fragments of telomeric DNA following replication [50] and, thus, telomere shortening [51], [52].

Starting from these assumptions, our interest was focused on the analysis of new biomarkers of chromosome instability, recently considered a new valuable tool to measure chromosome rearrangement and DNA damage [53] especially after telomere damage [25]. To our knowledge, no study demonstrated a direct relationship between telomere oxidation and the development of gross nuclear abnormalities. Therefore, the main aim of this work was to demonstrate a direct link between the two processes, i.e., telomeric regions are prone to DNA damage under oxidative stress and the persistence of base modifications in telomeres has an impact on nuclear abnormalities in human primary fibroblasts.

To assess the DNA damage induced by the acute oxidative stress treatment as well as to compare genomic and telomeric damage, we treated MRC-5 cells with two doses of hydrogen peroxide and we performed two different analyses – one on the whole genome by the FPG-modified alkaline comet assay and the other on telomeric sequences by the FPG-sensitive base lesions within telomeric DNA.

The analysis of the entire genome immediately (time 0) and 1 hr after treatment revealed an increase in damage that was completely rescued 24 hrs later. These results could be interpreted to signify that, as expected, the genomic damage was repaired within this time. In contrast, the analysis of telomeric sequences indicated an increase in DNA damage 1 hr after treatment that decreased over time but persisted at a significant level 24 hrs after treatment for both doses of hydrogen peroxide. The differences between the two techniques can be explained by considering that the comet assay highlights damage in the entire genome and not in the damage site specifically (e.g., telomeric fragments). The persistence of damaged bases in telomeric versus non-telomeric G-rich DNA suggests not only that the telomere structure is susceptible to oxidative stress but also that DNA damage repair at telomeres is less effective than in non-telomeric regions [54]. These results are in line with a previous study in which the authors demonstrated that acute oxidant exposure causes a high incidence of FPG-sensitive sites in telomeric DNA fragments [55]. These lesions persist at a higher level in telomeres after 6 hrs of recovery time, suggesting that the repair of oxidative DNA damage may be less effective in telomeres in vivo, most likely because the sequence context of telomere repeats and certain telomere configurations may contribute to the vulnerability of telomeres to the processing of oxidative DNA damage. Furthermore, unrepaired oxidative telomere damage can have undesired consequences during replication if not readily repaired.

With these assumptions, we focused our attention on telomeres to test whether persistent base damage has an impact on telomere length.

Our data revealed no telomere length modulation 24 hrs after treatment, while a significant telomere shortening was observed after 48 hrs. One likely explanation is that the persistent telomeric damage observed 24 hrs after treatment due to the 8-oxoG is not repaired and is responsible for the telomere shortening observed at 48 hrs, indicating that this phenomenon is dependent on replication. At the subsequent times, 72 and 96 hrs, we observed a restoration of telomere length, suggesting that telomere shortening is a transient consequence of acute oxidative stress.

Telomere shortening is a well-accepted cause of chromosome instability. For this reason, after demonstrating a change in telomere length, the next step was to investigate the relationship between the telomere shortening observed and chromosome instability through the analysis of biomarkers strictly related to telomere dysfunction such us MN, NBUDs and NPBs [25]. Especially for NPBs, we observed a dose-dependent increase 48 hrs after treatment. This result relates very well with the telomere shorting observed in the same time frame, allowing us to confirm the relationship between telomere shortening and the observed markers of chromosome instability. Because telomere shortening generated fusions of broken chromosome ends [15], [25], we believe that such fusions could induce an increase in observed chromosome bridges. Additionally, our analysis showed a decrease of NPBs at later times, which could be associated with the restoration of telomere length.

The strict correlation between NPBs and telomere length indicates that this biomarker represents a good readout for telomere defects and any consequent chromosome segregation errors. We hypothesised that telomere shortening leads to an increase in the rate of chromosome bridges; when telomere length is recovered, a decrease in the frequency of chromosome bridges is observed (Fig. 9).

To understand the mechanism leading to restored telomere length, we performed a telomerase activity assay to evaluate the possible involvement of this enzyme in the observed telomere modulation, and our results indicated no contribution. Furthermore, the ALT mechanism, a recombination mechanism between telomere sequences [56], was tested by CO-FISH (Chromosome Orientation-FISH) analysis, and the result was negative in this case as well. In our opinion, after acute oxidative stress, telomere restoration could be the result of a cellular selection system that promotes cells with longer telomeres due to their higher growth rate. Indeed, our results on cell viability and cell growth demonstrated that doubling time increased in treated cells, indicating that hydrogen peroxide treatment significantly reduced the MRC-5 proliferation rate. The idea is that cells with shorter telomeres could be responsible for the decreased growth rate, which is particularly noticeable in the log phase of the curve between 48 hrs, when telomeres are shortened, and 72 hrs, when telomere length is rescued. The results on cell viability did not indicate any differences between treated and control samples, allowing us to exclude an effect on cell viability in treated samples for our analysis times. This hypothesis could also explain the brief chromosome instability, which is restored in a short time.

In this work, we demonstrated a link between the oxidation process and abnormal nuclear morphologies. Oxidative stress has been shown to induce 8-oxoG at telomeric sequences. In addition, telomeres are repaired less efficiently than the whole genome [14], and the presence of base damage could interfere with the replication fork at the telomere, extending the portion of unreplicated ends [12] and disrupting the binding of shelterin protein [19]. The presence of unrepaired 8-oxoG can lead to GC to TA transversions after two rounds of replication [16]. Adenine could impede the binding of TRF1 and TRF2 to telomeres, interfering with their regulation of telomere length and telomere capping [19]. Together, these findings lead us to state that the persistence of 8-oxoG induces telomere shortening/dysfunction that causes telomeric fusions and gives rise to the accumulation of NPBs in cytokinesis-blocked cells. Moreover, according to other authors, NPBs should break at the subsequent cell division. This event could be responsible for the formation of MN (two breaks on the bridge) and NBUDs (one break on the bridge) [53], [25]. This corresponds well with our results in which we observed a delayed MN induction resulting from NPB breaks. We can conclude that oxidative stress leads to gross nuclear abnormalities by telomere shortening/dysfunction, and thus, telomere oxidation may be an instability-triggering mechanism.

It could be interesting to verify these results in other cells, i.e., tumour cells, to test whether telomerase activity can act as a protective mechanism against oxidative stress. Furthermore, to understand if protein alteration also contributes to the telomere shortening observed, the shelterin complex could be analysed.

Studies on acute oxidative stress exposition could be very useful considering that certain conditions may be responsible for increased oxidation levels, such as the tumour microenvironment, radiotherapy and some human diseases.
